# Anemia is associated with increased risk of contrast‑induced acute kidney injury: A Systematic Review and Meta-analysis

**DOI:** 10.1080/21655979.2021.1883887

**Published:** 2021-02-17

**Authors:** Wei Liang, Cheng Jie Yu, Qiong Ying Wang, Jing Yu

**Affiliations:** a Department of Cardiology, Lanzhou University Second Hospital, Lanzhou University, Lanzhou, China; b Medical Records Department, Lanzhou University First Hospital, Lanzhou University, Lanzhou, China

**Keywords:** Anemia, CI-AKI, coronary artery disease, meta-analysis

## Abstract

Previous studies have identified numerous risk factors of contrast-induced acute kidney injury (CI-AKI) in patients undergoing coronary angiography. However, the association between anemia and CI-AKI remains conflicting. Thus, we conducted a meta-analysis to further clarify the relationship between anemia and CI-AKI. PubMed, EMBASE and Web of Science were systematically searched from inception to June 2020 to identify eligible studies. The pooled odds ratios (ORs) with 95% confidence intervals (CIs) were used to estimate the correlation between anemia and CI-AKI. The potential publication bias was estimated using funnel plot and Begg’s test. A total of 13 studies (five case-control studies and eight cohort studies) comprising 27,135 patients were included. The pooled results showed that anemia was a significant risk factor of CI-AKI (OR, 1.82; 95% CI, 1.27–2.61). Moreover, the results of subgroup analyses and sensitivity analyses were basically consistent with the overall pooled result. Funnel plot and Begg’s test indicated that there existed potential publication bias, but the result of trim and filled analysis showed that the pooled results kept stable after adding ‘missing’ studies. This meta-analysis suggested that anemia may be correlated with an increased incidence of CI-AKI in patients undergoing coronary angiography. However, our conclusions should be interpreted with caution due to some limitations. Therefore, further high-quality trials should be conducted to confirm our findings.

## Graphical abstract

**Figure:**
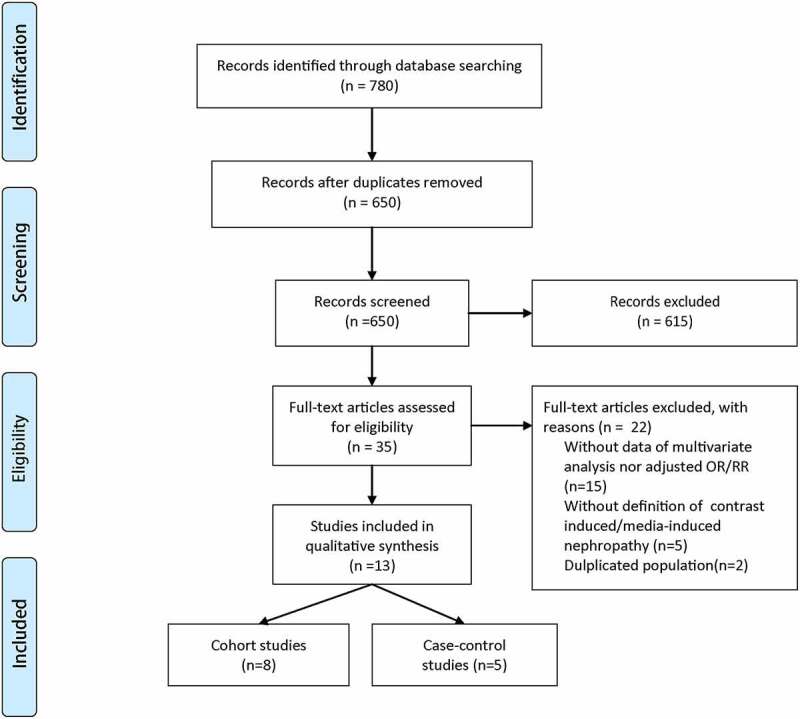

## Introduction

Coronary angiography has been widely applied in the diagnosis and therapy of coronary artery disease (CAD) for several decades [1]. Unfortunately, many clinical studies reported that coronary angiography could significantly increase the risk of contrast-induced acute kidney injury (CI-AKI) [1,2]. The reported incidences of CI-AKI were distinct across different studies, ranging from 2% to 30%. This inconsistency may be caused by the heterogeneous populations and different CI-AKI definitions [3,4]. CI-AKI has ranked as the third major cause of AKI in hospitalized patients [5,6]. Even worse, CI-AKI is a rather detrimental complication that closely correlates with high morbidity and mortality [7]. Therefore, it is very imperative to comprehensively identify risk factors of CI-AKI, which may help to establish the preventive strategies for CI-AKI.

Numerous risk factors of CI-AKI have been identified such as chronic kidney disease, hypertension, hyperuricemia, diabetes mellitus and older age, but most of these factors were irreversible [8,9]. To develop methods of reducing the incidence of CI-AKI, it is necessary and urgent to find potentially reversible risk factors of CI-AKI. Increasing evidence indicated an association between anemia and CI-AKI. On one hand, several studies suggested anemia was an independent risk factor of CI-AKI [9]. A few studies found no significant correlation between anemia and the incidence of CI-AKI [10,11]. These inconsistent results have left the relationship between anemia and CI-AKI in suspense, so further studies should be performed to resolve this issue.

The association between anemia and CI-AKI remains conclusive. Therefore, in this study we performed a meta-analysis of observational studies to systematically evaluate the correction between anemia and the incidence of CI-AKI, in order to provide the epidemiological evidence on this topic.

## Methods

### Literature search

We searched PubMed, Web of Science and EMBASE databases up to June 2020 using the following keywords: ‘anemia’ or ‘hemoglobin’ and ‘coronary angiography’ or ‘percutaneous coronary intervention’. Patients were searched by ‘anemia’ or ‘hemoglobin’; and ‘coronary angiography’, ‘percutaneous coronary intervention’ or ‘contrast’ for intervention; and ‘kidney failure’ for outcomes. Moreover, we screened the bibliographies of the relevant studies to identify additional articles.

### Selection criteria

The studies meeting all the following criteria were included in this meta-analysis [1]: observational study [2]; explored the relationship between anemia and CI-AKI in patients who underwent coronary angiography with or without percutaneous coronary intervention (PCI); and [3] reported outcomes with the adjusted odds ratios (ORs) or relative risks (RRs) with 95% confidence intervals (CIs). If more than one studies enrolled the overlapping population, we chose the newly published study. Meanwhile, we excluded studies in the form of review, comment, conference abstract or case report. Additionally, the language of publication was not restricted.

### Data extraction

Two investigators independently assessed the eligibility of all the studies according to the criteria mentioned above. If any disagreement occurred, they removed these issues through a deep discussion. A standardized form was used to extract the following variables from the retrieved studies: first author name, publication year, study design, country of study, period of research, the sex ratio of study population, the age of study population, the number of study population, definition of anemia, definition of CI-AKI, therapy (with or without PCI), outcome measure with the adjusted ORs or RRs with 95% CIs, and the adjusted variables.

### Assessment of methodological quality

We assessed the methodological quality of the included studies using the Newcastle-Ottawa Scale (NOS). In this system, three evaluative dimensions aspects were involved: selection of cases and controls, comparability between cases and controls and exposure in cases and controls. Scores from 0 to 9 may be given to a study based on the three dimensions. In the current study, we regarded more than mean score assigned to each study type as a cutoff to determine the quality.

### Statistical analysis

The ORs with 95% CIs were used to estimate the correlation between anemia and the incidence of CI-AKT. The heterogeneity across the included studies was calculated using the Higgins I^2^. The formula of calculating I^2^ is as follows: I^2^ = 100% x (Q-df)/Q, in which Q represents Cochran’s heterogeneity statistic and df stands for the degree of freedom. The value of I^2^ ranges from 0% to 100% and I^2^ value > 50% indicates a dramatical heterogeneity. Considering the unavoidable heterogeneity of observational studies, random-effects model was used to assess the pooled effect. Subgroup analyses were performed based on multiple stratification parameters, including study design type (case-control vs cohort, prospective vs retrospective), sample size, methodological quality, definition of anemia and region. Sensitivity analysis was performed by deleting single study in each step. Publication bias was assessed using Begg’s funnel plot. The p-value < 0.05 was considered to be statistically significant. All analyses were carried out using the Stata IC version 15.0 software package (StataCorp, College Station, Texas, USA).

## Results

### Study Selection

**Figure 1.**
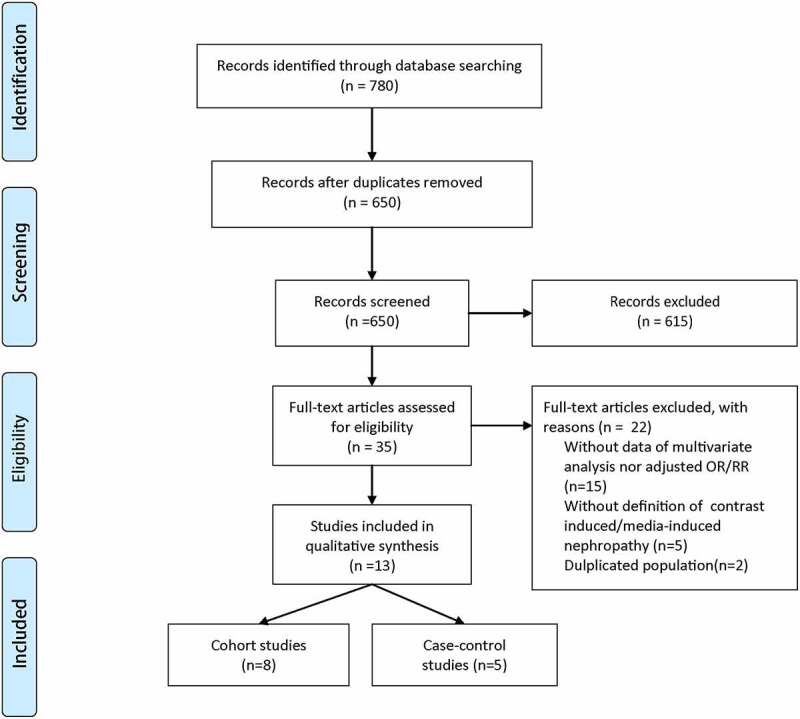
PRISMA flow diagram of literature selection

Relevant studies were identified as the flow diagram illustrated in Figure 1. A total of 780 articles were initially retrieved through systematically searching. After duplicates were removed, the remaining 650 studies were further screened by titles and abstracts, in which process 615 records were excluded for irrelevant topics. Then, a total of 35 articles were screened through full text. Among these 35 studies, we excluded 22 ones for the following reasons: 1) Fifteen studies did not report the results of multivariate analysis nor adjusted OR/RR; 2) Five studies were conducted without explicit definition of contrast-induced/media-induced nephropathy; and 3) Two studies included the duplicated patients [12,13]. Finally, 13 observational studies were included in the current meta-analysis, including five case-control studies [9,10,14–16] and eight cohort studies [8,11,13,17–21]. Of these articles, eight were conducted in Asia (two in China, two in Taiwan China, two in Japan, one in South Korea and one in Singapore), two in America and three in other countries (one in South Africa, one in Turkey and one in Israel).

### Characteristics of included studies

Baseline characteristics of the included studies are present in Table 1. A total of 13 studies included 27,135 patients with 9596 females and 17,539 males, were included. The sample size of the included studies ranged from 206 to 13,126. The definition of CI-CKI was almost same: CIN/CI-AKI was defined as serum creatinine level either 25% or 0.5 mg/dl from baseline values within 48 to 72 hours after contrast exposure [22,23]. Among those studies, eight studies defined anemia according to serum hemoglobin less than 11 g/dL to 13 g/dL, while Lie et al. [11] defined anemia as hematocrit < 39% in men and <36% in women. In addition, four studies did not report the definition of anemia (Table 2).

**Table 1.** General characteristics of the included studies

Author(et.al),Year	Country	Study design	Period of research	Population(F/M) age(years)	Definition of anemia	Definition of CIN	OR/RR(95% CI)	Adjusted variables	NOS score
Chong2010 [13]	Singapore	Retrospective cohort study	May 2000 to April 2008	3036(654/2382) 57.4	Anemia was defined as serum Hb<11 g/dL	CIN was defined as ≥25% or ≥0.5 mg/dL increase from baseline Cr within 48 hours after PCI	2.49(1.66–3.74)	Age group, gender, race, hypertension, anemia, low BP, LVEF 50, diabetes (no diabetes, noninsulin-dependent diabetes, insulin-dependent diabetes), GFR, CK500, race, and indication for PCI	8
Li2013 [16]	China	Hospital-based case-control study	1 January 2008 and 31 October 2009	1026(404/622) 64 (29–81)	Anemia was defined as hemoglobin<120 g/l in women and <130 g/l in men.	Contrast-induced nephropathy (CIN) was defined as the elevation of serum reatinine by ≥0.5 mg/dl or ≥25% occurring within 3 days after the intravascular administration of contrast medium, without an alternative etiology	2.352(1.395–3.453)	Age, sex, BMI, hypertension, hypercholesterolemia, LVEF, presence of diabetes mellitus, AMI, UAP, prior MI, baseline eGFR, amount of contrast agent administered, glucose level and hemoglobin level	7
Daisuke2014 [17]	Japan	Retrospective cohort study	April 2007 to April 2010	1954(443/1511) 69.1	Anemia was defined as a hemoglobin (Hb) level <10 g/dl/dl	CI-AKI was defined as an increase in serum creatinine of 0.5 mg/dl or 25% within 1 week from contrast-medium injection	2.31 (1.17–4.55)	Age, sex, CV/eGFR, prior CHF, multivessel disease, IABP, LVEF<40%, diuretic use, and Hb <10 g/dl	7
Kim2014 [10]	South Korea	Case-control study	September 2006 to December 2011	297(97/200)	NR	CI-AKI was defined as serum creatinine level either 25% or 0.5 mg/dl from baseline within 72 hours after contrast exposure	0.85(0.67–1.01)	Age, gender, body surface area, LV systolic dysfunction, clinical presentation, diabetes mellitus, type of contrast media, contrast V/CrCl >6.0, eGFR, serum hemoglobin, number of inserted stents, shock, PCI for left main (LM) coronary artery disease, and hydration before the procedure	6
Guo2015 [8]	China	Prospective cohort study	January 2010 to October 2013	1772(336/1436)	NR	Contrast-induced acute kidney injury was defined as an increase in serum creatinine of >0.5 mg/dL from the baseline within 48 to 72 hours of contrast exposure	1.959(1.036–3.704)	DM, males, LVEF<40%, emergent PCI, P_MI, age>60 mL/min/1.73 m^2^, diiuretic usage, hyperuricemia	7
Kurtul2015 [15]	Turkey	Case-control study	March 2012 to August 2014	814(256/558) 61 ± 12	NR	Contrast-induced acute kidney injury was defined as an increase in serum creatinine level of 25% or 0.5 mg/dL above the baseline value which occurs within 48 to 72 hours after the procedure	0.788 (0.650–0.956)	Age, women, diabetes mellitus, current smoker, heart rate, left ventricular ejection fraction, white blood cell count, hemoglobin, estimated glomerular filtration rate, total cholesterol, uric acid, creatine kinase myocardial band, high-sensitivity C-reactive protein, procalcitonin, SYNTAX score and total time of precedure	6
Shacham2015 [21]	Israe	Retrospective cohort study	January 2008 to December 2013	1248(237/1011) 61 ± 13	Anemia was defined as hemoglobin < 12 g/dL in women and < 13 g/dL in men, according to the World Health Organization criteria	AKI was determined using the AKI Network criteria, and defined as an sCr increase > 0.3 g/dL, compared with admission sCr	1.76(1.02–3.02)	Age, sex, hypertension, diabetes mellitus, left ventricular ejection fraction, admission eGFR, critical state, time to coronary reperfusion, and admission hemoglobin level or the presence of anemia	8
Banda2016 [18]	South Africa	Prospective cohort study	1 July 2014 to 30 July 2015	371(161/210) 49.3 (15.9)	Anemia was defined as serum hemoglobin (Hb) < 11 g/dL	CIN was defined as a serum creatinine increase of >25% from baseline or an absolute increase of 44 µmol/L assessed within 48–72 hours post contrast media administration as per the 2011 updated European Society of Urogenital Radiology (ESUR) guidelines	1.71(1.01–2.87)	Age, gender, albumin level and baseline eGFR	8
Hsieh2016 [14]	Taiwan, China	Case-control study	July 2003 to June 2015	377(108/269) 36.3 ± 17.4	Anemia due to acute bleeding (initial Hb < 11 g/dL)	CIN was defined as the relative (25%) or absolute (0.5 mg/dL) increase in serum creatinine within 48 h after contrast administration	3.16(1.46–6.81)	Body mass index, Injury Severity Score, Spleen Injury Scale, Large hemoperitoneum, Splenectomy, Splenectomy	7
Sato2016 [20]	Japan	Prospective cohort study	November 2011 to September 2013	853(198/655)	Anemia was defined by the World Health Organization criteria as a hemoglobin level < 13 g/dl for men and <12 g/dl for women	CIN was defined as increase in serum creatinine (SCr) ≥ 0.5 mg/dL or ≥25% from baseline between 48 and 72 h after exposure to contrast	1.94(1.08–3.61)	Age, male sex, diabetes mellitus, hypertension, CIN, SCr and anemia	9
Grossman2017 [19]	America	Prospective cohort study	1 January 2010 and 31 December 2013	13,126(6015/7111)	NR	CIN was defined as an increase in serum creatinine from baseline to post-PVI peak creatinine ≥0.5 mg/dLwas defined as an increase in serum creatinine from baseline to post-PVI peak creatinine ≥0.5 mg/dL	2(1.6–2.6)	A history of diabetes, anemia, CHF, and a pre-procedural CCC < 60 mL/min	8
Liu2017 [11]	Taiwan, China	Retrospective cohort study	February 2007 to September 2012	206(56/150) 65(55–77)	Anemia was defined as hematocrit < 39% in men and < 36% in women	CIAKI was defined as: 1) an absolute elevation of serum creatinine > 0.5 mg/dl in patients with baseline serum creatinine 2.0 mg/dl, or 2) a relative increase of 25% from the baseline value in patients with baseline creatinine > 2.0 mg/dl within 96 hours after primary PCI was performed.	0.908(0.689–1.197)	Age, creatinine, hemoglobin, multi-vessel disease,	8
Sreenivasan2018 [9]	America	Case-control study	January 2012 to December 2016	2055(631/1424) 58.0 ± 12.5	Anemia was defined as baseline hemoglobin≤13 g/dL. mild (11.1 to 13.0 g/dL), moderat (9.1 to 11.0 g/dL) and severe (7.0 to 9.0 g/dL)	Defined AKI as 0.5 mg/dL increase in serum creatinine from baseline following coronary angiography	5.3(3.8–7.3)	Race/ethnicity, prior CKD, prior heart failure, diabetes mellitus, hypertension, intra-aortic balloon pump (IABP) use prior to or within 24 hours of procedure, presence of cardiogenic shock, acute coronary syndrome (ACS)	7

CIN = contrast induced/media-induced nephropathy; CI-AKI = contrast-induced acute kidney injury; CM = contrast media; NR = not reported; CI = confidence interval; DM = diabetes mellitus; LVEF = left ventricular ejection fraction; OR = odds ratio; HR = hazard ratio; PCI = percutaneous coronary intervention; sCr = serum creatinine; P_MI = previous myocardial infarction; CHF = congestive heart failure; CCC = calculated creatinine clearance; BSA = Body surface area; eGFR = Estimated glomerular filtration rate; WHO = world health organization; CKD = chronic kidney disease; NOS = Newcastle–Ottawa Scale.

**Table 2.** Methodological quality of included studies based on the Newcastle–Ottawa Scale* for assessing the quality of case-control and cohort studies

Case-control studies (n = 4)	Selection	Comparability (control for important factors or additional factor)	Exposure	Total					
	Adequate definition of cases	Representativeness of cases	Selection of controls	Definition of controls		Ascertainment of exposure (blinding)	Same method of ascertainment for participants	Non-response rate	
Li2013 [16]	☆	☆	-	☆	☆☆	☆	☆	-	7
Kim2014 [10]	-	☆	☆	☆	☆☆	-	☆	-	6
Kurtul2015 [15]	☆	☆	☆	-	☆	☆	☆	-	6
Hsieh2016 [14]	☆	☆	☆	☆	☆☆	-	☆	-	7
Sreenivasan 2018 [9]	☆	☆	☆	☆	☆☆	-	☆	-	7
Cohort studies (n = 9)	Selection	Comparability (control for important factors or additional factor)	Outcome						
	Representativeness of exposed cohort	Selection of non-exposed cohort	Ascertainment of exposure	Outcome of interest not present at start of study		Assessment of outcome	Follow-up long enough for outcomes to occur^1^	Adequacy of follow-up of cohorts^2^	Total
Chong2010 [13]	☆	☆	☆	☆	☆☆	☆	☆	-	8
Daisuke2014 [17]	☆	☆	☆	☆	☆☆	☆	-	-	7
Guo2015 [8]	☆	☆	☆	☆	☆	☆	☆	-	7
Shacham2015 [21]	☆	☆	☆	☆	☆☆	☆	☆	-	8
Banda2016 [18]	☆	☆	☆	☆	☆☆	☆	☆	-	8
Sato2016 [20]	☆	☆	☆	☆	☆☆	☆	☆	☆	9
Grossman2017 [19]	☆	☆	☆	☆	☆☆	☆	☆	-	8
Liu2017 [11]	☆	☆	☆	☆	☆☆	☆	☆	-	8

*A study can be awarded a maximum of one star for each numbered item within the Selection and Exposure categories and maximum of two stars can be given for comparability. ^1^ A cohort study with a follow-up time > 6 months was awarded one star.

^2^A cohort study with a follow-up rate > 75% was awarded one star.

### Anemia and CIN

**Figure 2.**
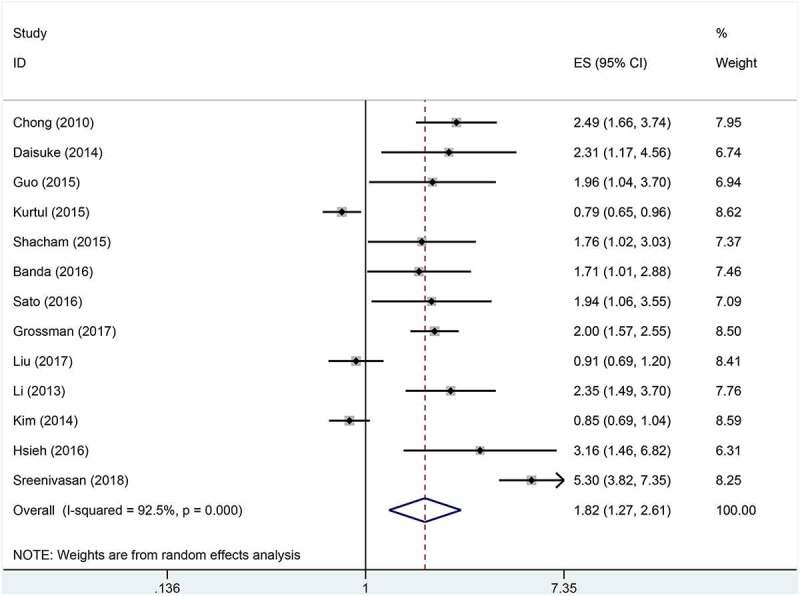
Forest plot of association between anemia and contrast-induced acute kidney injury (CI-AKI) (OR, 1.82; 95% CI, 1.27–2.61)

**Figure 3.**
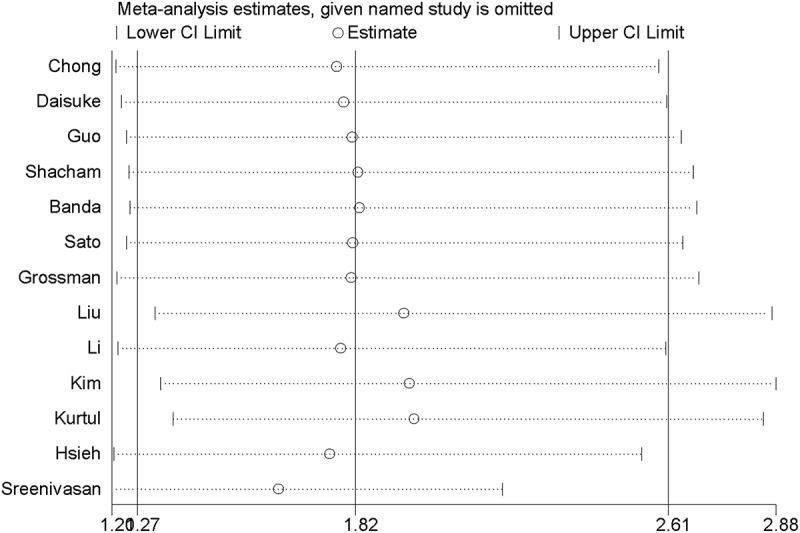
Sensitivity analysis of association between anemia and CI-AKI: the result showed the pooled ORs were stable

The overall pooled result indicated that anemia was associated with increased risk of CI-CKI (pooled OR = 1.82, 95% CI 1.27–2.61), but it was accompanied by a high heterogeneity across the included studies (I^2^ = 92.5%) (Figure 2). Subsequently, subgroup analyses (Table 3) were conducted to explore the sources of statistical heterogeneity. The pooled OR of eight cohort studies was 1.77 (95%CI = 1.32–2.38), with evidence of moderate heterogeneity (I^2^ = 73.0%). However, the pooled result of case-control studies showed anemia was not significantly related to CI-AKI (pooled OR = 1.88, 95%CI = 0.89–3.96). In the subgroup analysis based on study design, the pooled OR/RR of four prospective studies was 1.95 (95% CI = 1.6–2.37), with no evidence of interstudy heterogeneity (I^2^ = 0), indicating an association between anemia between high incidence of CI-AKI. Similarly, the pooled result of nine retrospective studies also suggested anemia was correlated with increased risk of CI-AKI (pooled OR = 1.79, 95%CI = 1.11–2.89; I^2^ = 94.5%). In the subgroup analysis by sample size, the pooled result of seven studies with large sample (N ≥ 1000) indicated a significant association between anemia and CI-AKI (pooled OR/RR = 2.48, 95%CI = 1.78–3.47), with moderate interstudy heterogeneity (I^2^ = 76.8). However, no statistical significance was observed for the pooled analysis of six studies with small sample size (N < 1000) (pooled OR/RR = 1.17, 95% CI = 0.87–1.58). The pooled results of six studies with high quality (score ≥ 8) (pooled OR/RR = 1.70, 95%CI = 1.19–2.42; I^2^ = 79.6%) and seven studies with low quality (score < 8) (pooled OR/RR = 1.94, 95%CI = 1.05–3.58; I^2^ = 95.3%) suggested there was a close relationship between anemia and CI-AKI. In the subgroup analysis by the definition of anemia, the pooled result of eight studies defining anemia according to the serum hemoglobin (pooled OR/RR = 2.20, 95% CI = 1.21–4%), with evidence of high interstudy heterogeneity (I^2^ = 93.9%), indicated a potential relationship between anemia and CI-AKI. Nevertheless, no significant association between anemia and CI-AKI was found in the pooled analysis of five studies with other definition of anemia (pooled OR/RR = 1.35, 95% CI = 0.88–2.07%; I^2^ = 88.9%). Additionally, we further performed a subgroup analysis based on study region and found an association in Asia (pooled OR = 1.74, 95%CI = 1.17–2.59; I^2^ = 86.0%) and America (pooled OR = 3.24, 95%CI = 1.24–8.41; I^2^ = 85.0%), but no relationship in other countries (pooled OR = 1.28, 95%CI = 0.69–2.37; I^2^ = 95.5%). Then, sensitivity analysis was performed to evaluate the stability of the overall pooled results. As shown in Figure 3, the pooled results kept stable basically after omitting one included study each time. Overall, the pooled results suggested there was a correlation between anemia and CI-AKI. Although significant heterogeneity existed, our subgroup analyses and sensitivity analysis supported the robustness of the pooled results.

**Table 3.** Subgroup analysis of association between anemia and contrast media-induced nephropathy based on various factors

Outcomes	Number of trials	OR/RR (95% CI)	Heterogeneity, I^2^ (%)
Pooled results	13	1.82 (1.27–2.61)	92.5
Subgroup analyses based on study type			
Cohort studies	8	1.77 (1.32–2.38)	73
Case-control studies	5	1.88 (0.89–3.96)	96.8
Subgroup analyses based on study design			
Prospective studies	4	1.95 (1.60–2.37)	0
Retrospective studies	9	1.79 (1.11–2.89)	94.5
Subgroup analyses based on sample size			
N ≥ 1000	7	2.48 (1.78–3.47)	76.8
N < 1000	6	1.17 (0.87–1.58)	79.3
Subgroup analyses based on quality of included studies (NOS)			
≥8	6	1.70 (1.19–2.42)	79.6
<8	7	1.94 (1.05–3.58)	95.3
Subgroup analyses based on definition of anemia			
Hb level	8	2.20 (1.21–4.00)	93.9
NR or Others	5	1.35 (0.88–2.07)	88.9
Subgroup analyses based on rergion			
Asia	8	1.74 (1.17–2.59)	86
America	2	3.24 (1.24–8.41)	85
Other	3	1.28 (0.69–2.37)	95.5

OR = odds ratio; RR = relative ratio; CI = confidence interval; Hb = hemoglobin; NOS = Newcastle-Ottawa Scale; NR = not report.

### Quality of Evidence and Publication Bias

**Figure 4.**
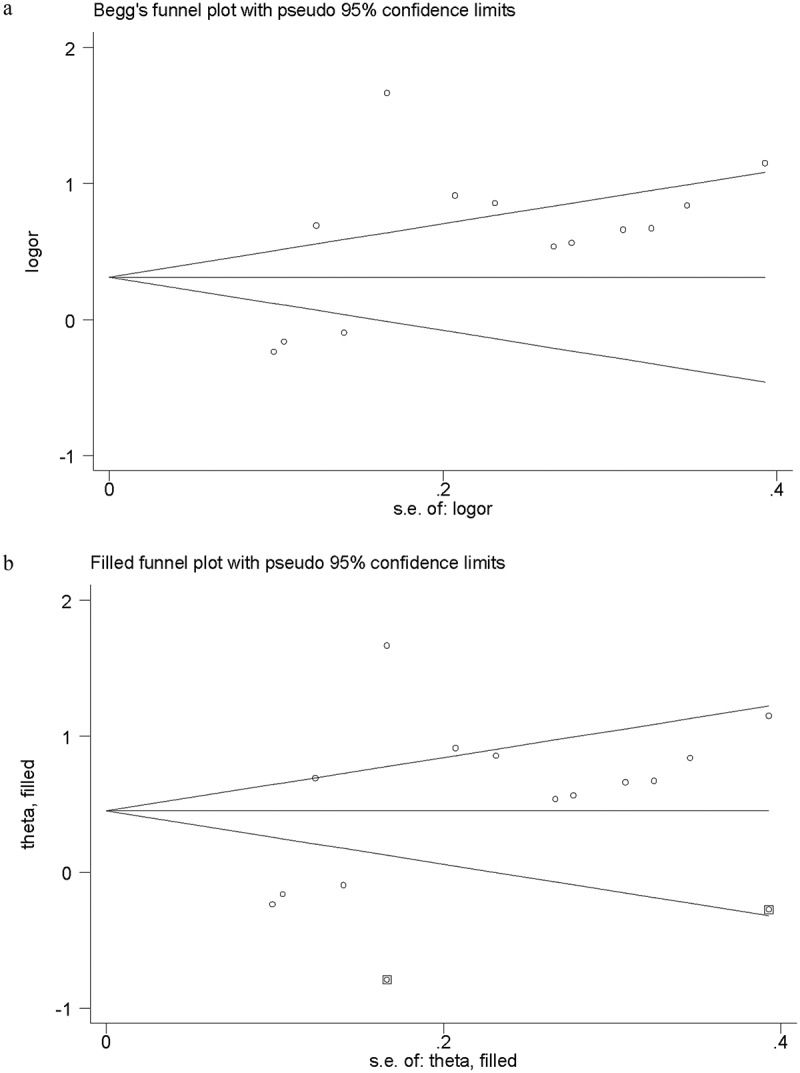
Primary funnel plot for publication bias (a) (Egger`s test: P = 0.045); Adjusted funnel plot from trimming estimator and filled analysis for publication bias (Pooled OR, 1.57;95% CI, 1.10–2.25, P = 0.013)

The methodological quality of eligible articles was assessed based on NOS criteria. The scores of case-control studies ranged from 6 to 7, and the scores of cohort studies ranged from 7 to 9. A definite cutoff of 8 was applied to determine the quality of a study and six studies were considered high quality (score≥8). The Funnel plot was performed to identify publication bias and its asymmetry was recognized from visual inspection, Then, trimming estimator and filled analyses were applied to analyze and the pooled estimate result was relatively stable (Figure 4).

## Discussion

In this meta-analysis, the overall pooled result suggested that anemia might be associated with an increased incidence of CI-AKI in patients undergoing coronary angiography. Moreover, the results of subgroup analyses and sensitivity analysis were basically consistent with the overall pooled estimate.

Increasing studies showed that CI-AKI has a strong link with adverse clinical outcomes, including cardiovascular complications, renal failure, prolonged hospitalization and death [24]. With the incidence of CI-AKI increasing, over 80 million studies were conducted worldwide to identify the potential risk factors [25]. Numerous risk factors of CI-AKI including history of chronic kidney disease, older age, cardiovascular disorder, diabetes mellitus, higher volume of contrast medium, hypotension and shock have been reported [26,27]. As a frequent feature of kidney injury, anemia might be a potential risk factor for chronic kidney disease [28]. However, the association between anemia and CI-AKI remains controversial. In the current study, our overall pooled estimate showed anemia was a risk factor of CI-AKI (pooled OR/RR = 1.82, 95%CI = 1.27–2.61) with or without impaired renal function before contrast medium exposure. Moreover, subgroup analysis based on study design and type, sample size, methodological quality, definition of anemia and region found similar findings. Notably, the subgroup analysis of studies defining anemia based on the serum hemoglobin suggested that there was an association between anemia and CI-AKI, whereas no significant association between anemia and CI-AKI was found in the pooled analysis of five studies with other definition of anemia, which was consistent with the conclusion of Liu et al. [11]. One possible explanation for this phenomenon might be the heterogeneity across the included studies. The same result was obtained among other regions, including South Africa, one in Turkey and one in Israel. However, an association between anemia and CI-AKI was more significant in America than Asia, which was consistent with the conclusion by Sreenivasan et al. [9] that African American patients were more likely to suffer CI-AKI. This difference might be explained by the fact that the hemoglobin level of healthy blacks is lower than whites in America [29].

Several possible biological mechanisms were considered to explain the association between anemia and CI-AKI. Kidney is a kind of highly oxygen-sensitive organ, so decrease in oxygen transport of blood, reduced blood volume, insufficient effective circulation and blood dilution would increase the consumption of oxygen and injury of oxidative stress in renal tubules cells [30]. Besides, studies have shown that decreased renal perfusion pressure, activation of inflammatory response factors and formation of small thrombi can lead to renal ischemia reperfusion injury [31], which was also verified by animal experiment [32]. More importantly, anemia also increases oxygen free radical damage and imbalance of vasoactive substances, which are able to promote apoptosis and immune injury of kidney cells [33–35]. Therefore, considering the potentially important role of anemia in the onset and development of CI-AKI, clinical workers and doctors should ensure oxygen supply and correct anemia in high-risk patients.

Our study also had several limitations. First, there existed significant heterogeneity across included studies, which might impair the authenticity of pooled effect. However, the results of subgroup analyses and sensitivity analyses were basically consistent with the overall pooled estimate, suggesting that our overall pooled result was robust and reliable. Second, the visual inspection of funnel plot indicated there existed potential publication bias in the meta-analysis, irrespective of the fact that we performed a comprehensive literature search. Interestingly, the result of trim and filled analysis showed that the pooled results kept stable after adding ‘missing’ studies, which indicated that the publication bias might not substantially affect the robustness of our pooled result. Third, although most of the included studies made the definition of anemia on serum hemoglobin level, the cutoff values were not consistent. Moreover, information about the types of anemia was not provided in most eligible studies, so it was hard to exclude the possibility that the pathologies of anemia might be different. Obviously, these potential differences across the included studies may bias our pooled results. Further studies are needed to evaluate the association between anemia and CI-AKI according to different serum hemoglobin level, such as hemoglobin≤13 g/dL, mild (11.1 to 13.0 g/dL), moderate (9.1 to 11.0 g/dL) and severe (7.0 to 9.0 g/dL), as well as the different types of anemia. At last, numerous factors have been considered to be correlated with the incidence of CI-AKI. In this meta-analysis, we found that anemia was a risk factor of CI-AKI, but we could not determine whether anemia is the most important. To ascertain which risk factor is the paramount for predicting CI-AKI, more multi-center clinical studies with large sample size may be performed and multiple credible algorithms should be applied to analyze the relevant data.

## Conclusion

To sum up, our findings suggested that anemia might be associated with an increased incidence of CI-AKI. However, the conclusion should be interpreted with caution due to some potential confounding factors and heterogeneity. Therefore, further high-quality trials should be performed to further confirm our findings.

## Highlights

1. Anemia may be correlated with CI-AKI.

2. Association between anemia and CI-AKI may vary from ethnicity.

3. Association between anemia and CI-AKI may vary from the definition of anemia.
